# Robin: an advanced tool for comparative loop caller result analysis leveraging large language models

**DOI:** 10.1093/nargab/lqag009

**Published:** 2026-01-30

**Authors:** H M A Mohit Chowdhury, Mattie Fuller, Oluwatosin Oluwadare

**Affiliations:** Department of Computer Science and Engineering, University of North Texas, 1155 Union Circle, Denton, TX 76203, United States; Center for Computational Life Sciences, University of North Texas, Denton, TX 76207, United States; Department of Computer Science, University of Colorado at Colorado Springs, 1420 Austin Bluffs Pkwy, Colorado Springs, CO 80918, United States; Department of Computer Science and Engineering, University of North Texas, 1155 Union Circle, Denton, TX 76203, United States; Center for Computational Life Sciences, University of North Texas, Denton, TX 76207, United States; Department of Computer Science, University of Colorado at Colorado Springs, 1420 Austin Bluffs Pkwy, Colorado Springs, CO 80918, United States

## Abstract

There has been significant interest in genomics research, leading to the development of numerous new methods. One notable area of progress is in chromosome loop detection algorithms (also known as loop callers). However, despite these advancements, there is no available platform to analyze, compare, or benchmark current tools’ results on the go. Developing such a platform is crucial to accelerating research and ensuring the reliability and effectiveness of new methods in this field. Hence, in this work, we propose Robin, an advanced, ready-to-go platform for comparative loop caller result analysis that leverages large language models (LLMs). Robin is a web server designed to analyze loop caller results, offering a comprehensive range of analysis metrics, such as recovery and overlap. It is tightly integrated with HiGlass for interactive, multi-resolution visualization of Hi-C matrices and loop annotations, allowing users to visually inspect and validate loop structures in a genomic context. Additionally, Robin incorporates LLM capabilities that enable users to generate customized plots and figures simply by providing natural language instructions. Overall, Robin is a robust and comprehensive loop caller result analysis and visualization tool. It is publicly accessible at http://hicrobin.online, with comprehensive documentation available at http://documentation.hicrobin.online/.

## Introduction

Chromosomes form complex and tightly confined structures that package DNA within small nuclear compartments [1]. To achieve this compact organization while maintaining regulatory accessibility, the genome forms loops that arise from both intra- and inter-chromosomal interactions. These chromatin loops facilitate regulatory communication and contribute to gene expression control [2–4]. Loop formation is mediated by key architectural proteins such as CTCF and the cohesin complex, which act together to establish and stabilize chromatin interactions [5]. CTCF serves as both an insulator protein and a transcriptional regulator, while cohesin helps with loop extrusion and maintenance. These proteins provide essential structural and functional support to the 3D genome. Additionally, epigenetic markers such as H3K27ac, a histone modification associated with active enhancers, play a role in distinguishing active regulatory regions from poised or inactive ones [6]. Together, these features help define biologically meaningful loop regions, which have drawn increasing scientific attention due to their diverse regulatory roles and significance in genome organization.

There are many loop caller tools available, such as cLoops [7], Mustache [8], and HiCCUPS [9], each with diverse input parameters and accepting different types of input data formats, such as Hi-C [4], ChIA-PET [10], etc. Researchers often benchmark their tools by considering various use cases and employing different types of metrics. This process typically involves writing extensive boilerplate code, and there is currently no platform available to evaluate the performance of each tool comprehensively. To address this gap, Chowdhury et al. (2024) published the first comparative study of loop callers [11]. In their study, they analyzed and categorized 22 different types of loop callers and benchmarked 11 of these tools side by side. Their study also introduced a novel Recovery Efficiency Metric (REM) score to demonstrate the tools’ efficiency in recovering specific proteins. This metric evaluates the performance of each loop caller by focusing on the recovery rate and normalizing it by the number of loops. This ensures fair, independent assessments of loop calling algorithms and prevents bias from tools that detect an excessive number of loops [11].

Despite the valuable insights from this study [11], the analysis remains static and customized to the tools considered, leaving current and future tool analyses unsupported. Given the rapid growth in this field, there is a pressing need for a dynamic, on-the-go analysis tool that provides a comparative platform for ongoing research and supports the future development of loop-calling methods. To address this need, we developed Robin, an advanced, ready-to-go platform for comparative loop caller result analysis and visualization leveraging large language models (LLMs). Robin is a web server designed to analyze loop caller results, providing a comprehensive range of analysis metrics such as consistency, recovery, and regression analysis. Additionally, Robin is integrated with HiGlass [12], a powerful web-based visualization framework for genomic interaction maps, enabling users to interactively explore and visualize loop caller outputs in a multi-resolution and scalable manner. This integration enhances interpretability and provides a user-friendly interface for visual validation of chromatin loops within their genomic context. Robin leverages an LLM to facilitate the generation of user-specific plots with ease. Furthermore, users can download high-resolution plots directly from the Robin web server for detailed analyses.

## Materials and methods

Robin has five main features: overlap analysis, regression analysis, recovery analysis, HiGlass visualization, and an AI-powered data visualization assistant that leverages LLMs to generate new graphs at runtime and based on users’ preferences beyond the analysis plots we provide. The overlap analysis captures the number of common loops across three different loop callers’ results in the form of a Venn diagram. It demonstrates computational consistency among different loop callers. The regression analysis identifies tools that fall within or near the regression boundary. This analysis is calculated between loop size (in kilobases) versus resolution, and loop size (in number of bins) versus resolution. In both cases, resolution is treated as the dependent variable and size as the independent variable. The regression analysis shows both categorical (if categories were defined by the user) and linear regression of the average loop size reported by each loop caller (in kb and number of bins) versus resolution.

In this work, protein recovery plots display the recovery rate and REM analysis, along with graphs comparing results at high resolutions (5 kb, 10 kb) and low resolutions (100 kb, 250 kb). The recovery rate reports the biological consistency of a loop caller. We calculated the recovery rate [11], $\delta$, for every thousand loops


(1) \begin{eqnarray*}
\delta = \frac{\#N_o}{\#N_{ref}},
\end{eqnarray*}

where $\#N_{ref} =$ the number of records in the reference file (e.g., CTCF, H3K27ac, RNAPII), and $\#N_o =$ the number of overlaps. Here, the overlap is calculated between a reference file and a loop file and uses a window (in our analysis, we used a 50-base-pair window size). REM measures the performance of every loop caller using the recovery rate, $\delta$, and ensures unbiased results regardless of any parameters, such as loop counts. Chowdhury et al. [11] defined REM, $\Delta$


(2) \begin{eqnarray*}
\Delta = \frac{\delta }{\#N_c},
\end{eqnarray*}

where $\delta =$ the recovery rate, $\#N_c =$ the loop count. The consistency score is calculated using REM between high and low resolutions from different biological features (e.g. CTCF, H3K27ac, RNAPII). Chowdhury et al. [11] defined the consistency score, $\Lambda$


(3) \begin{eqnarray*}
\Lambda = |\bar{\chi }_{\mathrm{low}} - \bar{\chi }_{\mathrm{high}}|,
\end{eqnarray*}

where $\bar{\chi }_{\mathrm{low}} = \Delta$ for low resolutions (e.g. 100 kb, 250 kb) and $\bar{\chi }_{\mathrm{high}} = \Delta$ for high resolutions (e.g. 5 kb, 10 kb). It is worth noting that users can customize and define their own high- and low-resolution ranges based on their specific analysis needs. Robin’s HiGlass integration automatically processes all uploaded result files and protein reference files provided by the user.

Additionally, Robin uses OpenAI’s GPT model [13] as a backend to generate scripts, presenting users with a Jupyter Notebook containing all resulting graphs and source code during execution. To enhance flexibility, we also provide a manual download option within the AI Assistant interface, allowing users to download analysis files in *.json* format and generate customized plots using their preferred Generative AI (GenAI) platform. This feature further enhances the robustness and adaptability of our tool beyond its current capabilities.

### Core architecture

The core architecture of Robin consists of a few parts: (i) frontend, (ii) web-api or backend, and (iii) database. All three of these components are bundled together in various Docker images, and they are connected in the same network and communicate with each other using the docker-compose command. The frontend is one of the main parts of Robin, as users directly interact with this part. We used React with React-Bootstrap and used React-Styleguidist to generate an isolated development environment for each component and build developer documentation. In this way, Robin’s web server was developed to be modular and scalable, with most components being developed in isolation, thus making side effects or unintended state changes less likely. We used React for its diverse features, and it is also maintainable. We used chart.js to render different types of plots. The web API handles all interactions among the frontend, database, and HiGlass, such as submitting jobs, retrieving data, communicating with AI assistants, and updating job information, etc. The web API is data-driven and executes analysis scripts to generate job results, acts in response to requests from the database, and runs Jupyter Notebook. We used Python, R, and bash scripts to calculate loop size, overlap, and recovery analysis. We wrote a worker that handles HiGlass and that converts and prepares data using bedtools and HiGlass Clodius to make them compatible with the HiGlass environment. We implemented HiGlass and the main Robin server in different Docker containers, and Robin has the capability to communicate internally among servers.

### LLM system

LLM is implemented in Robin’s AI Assistant by utilizing OpenAI’s model to generate Python code. The generated code is stored in the browser’s memory, allowing users to review and verify the AI-generated code. While the AI-generated Python code serves as an assistant, users are encouraged to thoroughly review it for correctness, as human oversight remains essential for catching potential logical errors. Once the user finds the generated code satisfactory and submits it, the code is sent to the Robin API and executed in a container running a Flask API to ensure it runs without any exceptions. After validation, the code is written to a Jupyter Notebook file, which is compiled and served to the end user as an embedded HTML in the results section. These notebook files are stored in association with the job on Robin’s server, allowing all results to be viewed at any time.

## Results

**Figure 1.**
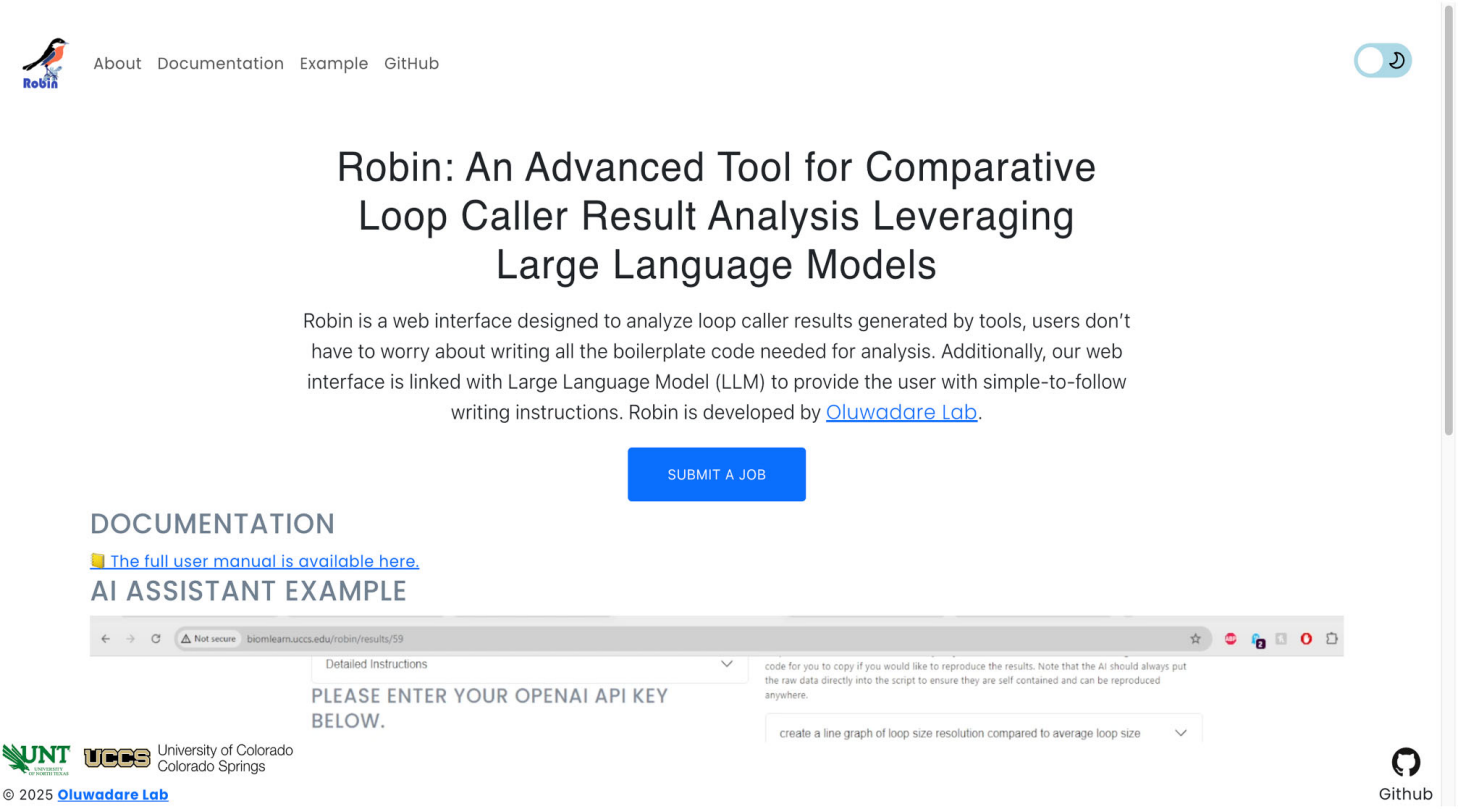
Robin web server landing page for loop caller result analysis. From this page, users can navigate to different options, such as submitting a job, reading documentation, etc.

Robin is a web server for comparative loop caller result analysis leveraging an LLM model (Fig. 1). Users can upload their loop caller results into Robin and export the analytical result plots in high resolution. It is integrated with HiGlass [12], a web-based platform for visual exploration and comparison of Hi-C genome interaction maps and other genomic tracks. We have seamlessly integrated HiGlass for Robin users, streamlining the process and making it easy to use. We utilized Robin to evaluate the results of 11 state-of-the-art loop callers [7, 8, 14–22]. Using these loop callers, we predicted chromatin loops from the Human Lymphoblastoid (GM12878) cell dataset [9, 23] at 5, 10, 100, and 250 kb resolutions. The results from this analysis are presented as an example on Robin’s web server and are further discussed in the following section.

### Robin analysis result on GM12878 dataset

First, using the GM12878 dataset for all chromosomes at 5, 10, 100, and 250 kb resolutions, we predicted loops using 11 loop callers. For ease of use, we have provided Dockerized container environments that include each of these tools along with the necessary parameter scripts, allowing users to run them with their desired configurations. Once the loop results are generated, they can be uploaded into Robin for downstream analysis. Next, to use the loop callers’ results on Robin, the outputs must be in the *.bedpe* format, which is the standardized format used by many loop callers to represent loop regions. We have provided a detailed description of this format in our documentation, along with example data generated by several state-of-the-art loop callers. Finally, once the loop caller outputs are confirmed to be in the correct format, they can be uploaded for use on the Robin web server. This process was followed for the GM12878 dataset at different resolutions, and the resulting analyses can be viewed on Robin’s example page.

**Figure 2.**
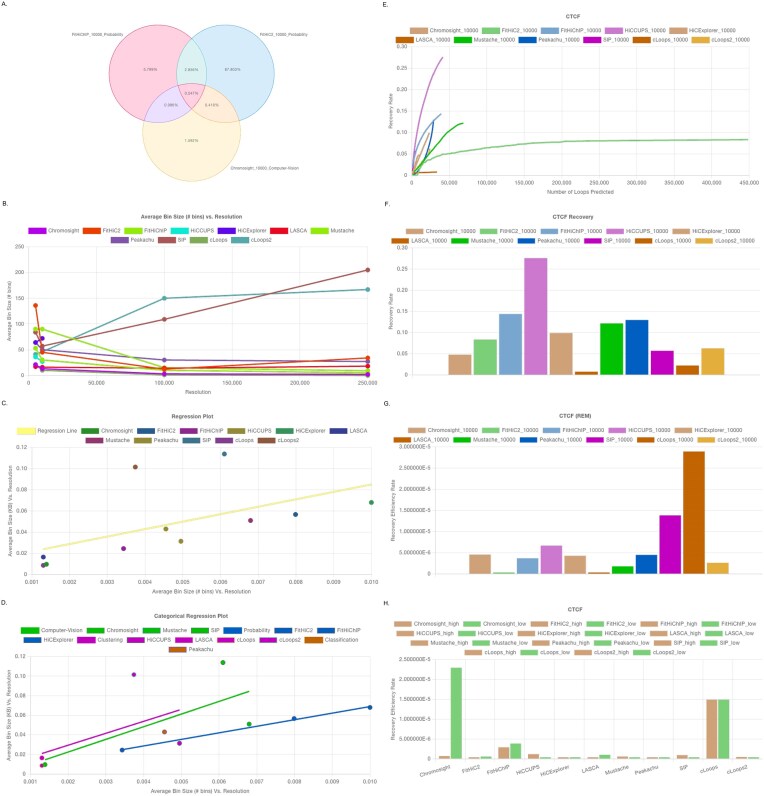
Result analysis using different loop-caller results using GM12878 for all chromosomes at high (5 and 10 kb) and low (100 and 250 kb) resolutions. (**A**) Robin’s overlap tab depicts the common loops across different loop callers. Robin’s regression tab contains: (**B**) average bin size versus resolution, (**C**) overall regression plot, and (**D**) categorical regression plot. Robin’s protein-specific tabs are named according to the uploaded protein reference file and contain the following: (**E**) recovery rate versus number of loops line plot, (**F**) recovery rate bar plot, (**G**) REM plot, and (**H**) REM plot between high and low resolution.

The first tab of results reports on the detected overlaps (Fig. 2A). This overlap illustrates the percentage of commonly identified loop regions, highlighting the consistency of loop callers across up to three tools in a single plot. Users can navigate through different resolutions. Next, the regression analysis tab includes plots of average bin size (number of bins and size in kb) versus resolution (Fig. 2 B). These plots show how bin size varies with resolution and display the average number of bins at each resolution. This tab also contains regression plots—both overall and categorical—to demonstrate the consistency of the loop callers (Fig. 2C and D).

Following the regression analysis, the protein recovery tab presents plots shown in Fig. 2 E–H. Recognizing the biological significance of chromatin-associated proteins, we provide protein-specific recovery rate analyses (e.g. for CTCF, H3K27ac, and RNAPII) based on user input and computed using equation (1). In the protein recovery line plot (Fig. 2 E), the *x*-axis represents the number of predicted loops, segmented into intervals of 1000. The *y*-axis indicates the recovery rate, which increases with each additional 1000 predicted loops. Selecting any point on the curve reveals the recovery rate based on the first *x* loops predicted by a tool, while the endpoint shows the total recovery rate across all predictions. We also include a bar plot illustrating the total recovery rate for each tool (Fig. 2 F). Additionally, we incorporate a REM plot (computed using equation (2)) to quantify recovery efficiency relative to the number of detected loops (Fig. 2 G).

To assess consistency across resolutions, we provide a comparison plot (computed using equation 3), which contrasts high-resolution (5 and 10 kb) and low-resolution (100 and 250kb) REM scores averaged per tool (Fig. 2H). We demonstrate this feature using three proteins: CTCF, H3K27ac, and RNAPII, in our example analysis. However, users may upload additional protein datasets, and the platform will generate corresponding tabs for each.

HiGlass integration provides ChIP signal plots for individual tools, enabling users to visualize loop positions relative to known protein-binding sites. This feature aids in validating recovery analyses. Finally, the “Data” tab displays the uploaded input files and provides a download option for the user’s convenience.

Running time is one of the most crucial factors for a tool’s usability. Hence, to evaluate the runtime performance of analysis tasks on Robin, we conducted a runtime analysis using output from the 11 different loop-calling algorithms across various resolution scenarios. Table 1 presents the time (in seconds) required to complete the analysis and generate results on Robin using the GM12878 dataset. Across all analysis types and options for the 11 algorithms, we observed that Robin achieves an average execution time of 88.75 seconds. This indicates that Robin provides an efficient turnaround for downstream analysis and visualization, making it well suited for rapid, iterative comparisons in chromatin loop detection workflows.

**Table 1.** Robin run-time analysis using GM12878 for all chromosomes at different resolutions

Set	Time (seconds)
5 and 10 kb	90
5 and 100 kb	80
100 and 250 kb	82
5, 10, 100, and 250 kb	103
**Average**	**88.75**

We recorded the running time in seconds using a different set of result files and calculated the average running time.

### Quantitative explanation of Robin’s analysis result

We also performed a quantitative analysis of the tools’ results to assess their performance. To do this, we adopted the quantitative scoring framework introduced by Chowdhury et al. [11], known as the $\mathrm{ BCC}_{\mathrm{ score}\mathrm{ }}$, which evaluates a tool’s performance across three dimensions: biological, consistency, and computational scores. This scoring system provides flexibility, enabling users to assign weights based on the importance of each feature in their specific use case. Chowdhury et al. [11] defined the $\mathrm{ BCC}_{\mathrm{ score}}$ as follows:


(4) \begin{eqnarray*}
\mu = \frac{\sum _i W_i X_i}{\sum _i W_i}
\end{eqnarray*}

where $\mu = \mathrm{ BCC}_{\mathrm{ score}}$, $i = [1, 2, 3]$ corresponds to biological, consistency, and computational feature scores, respectively, $X_i$ is the individual feature score, and $W_i$ is the assigned weight. The resulting $\mathrm{ BCC}_{\mathrm{ score}}$ ranges from 0 to 1, with higher values indicating better performance.

To quantify the effectiveness of Robin’s analysis, we computed the $\mathrm{ BCC}_{\mathrm{ score}}$ using the GM12878 dataset across all chromosomes at 10 kb resolution. For this computation, we considered only the biological and consistency features, as these metrics can be directly extracted from Robin’s output. The consistency score was derived by aggregating the overlap percentages of each tool with two additional algorithms, yielding five triplet comparisons per tool. The reported consistency score is the average score for each algorithm across all these triplet comparisons (see Supplementary Data 1, Overlap Tab). The biological feature score was calculated by averaging the REM scores from each loop caller for CTCF, H3K27ac, and RNAPII, as reported by Robin (see Supplementary Data 1, Biological Tab).

As the $\mathrm{ BCC}_{\mathrm{ score}}$ is a weighted metric, we assigned a weight of 1 to consistency and 2 to biology, reflecting our belief that biological relevance is more critical in loop detection. Based on this scoring, we observed that cLoops [7] achieved the highest $\mathrm{ BCC}_{\mathrm{ score}}$ of $15.79 \times 10^{-2}$ among the 11 evaluated loop callers (see Table 2).

**Table 2.** Different tools’ performance comparison using Robin

Tool	Consistency (%)	Biological (%)	$\mu$ ($\times 10^{-2}$)
LASCA	0.2714	0.3176	0.4831
cLoops	1.2360	21.8354	15.7929
cLoops2	1.9832	3.2262	4.1340
HiCCUPS	6.0684	6.7759	10.5857
HiCExplorer	4.0634	3.8357	6.6205
FitHiC2	0.1634	0.4064	0.4343
FitHiChIP	2.8674	5.6856	6.6578
Peakachu	3.9576	4.1083	6.6965
Mustache	2.8582	1.5807	3.9120
Chromosight	1.6618	6.3758	5.9123
SIP	3.0936	7.7147	8.2367

Biological and consistency scores were measured using GM12878 for every chromosome at a resolution of 10 kb. Overlap percentage is used as the consistency score, and protein (CTCF, H3K27ac, RNAPII)-specific REM scores are considered as the biological score in this analysis. We calculated BCC_score_ using consistency and biological scores, and cLoops achieved the highest score. The best result is in bold.

### Hybrid analysis: user-supervised LLM-generated code

Leveraging the LLM (OpenAI GPT) allows the user more freedom and options for quick data analysis and visualization. The AI assistant included in the results tab (Fig. 2E) allows the user to prompt the AI system to generate new graphs to visualize data analysis that is not shown in Robin. This process begins with instructing the AI to generate code snippets for performing a desired analysis based on the data uploaded by the user. Once the code is generated, the user reviews and supervises the code to ensure logical correctness, making any necessary updates before submitting it to be executed within Robin. All AI-generated code is fully self-contained, including any referenced data, enabling users to edit scripts directly on the Robin platform or download them for external use without requiring additional data files. To further support flexible downstream analysis, users can also download the associated analysis file, enabling them to export and visualize results using their own GenAI tools. This enhances the overall versatility and reproducibility of Robin’s analysis.

As previously mentioned, user supervision is a crucial component of this process. While LLMs can generate code effectively, it is important to recognize that they may occasionally produce results containing errors or with missing logical components. Therefore, while the AI Assistant is a valuable tool for rapidly prototyping data visualizations, we strongly advise users to thoroughly review and supervise the generated code, using it for exploratory analysis. With appropriate user oversight, this tool can greatly facilitate quick and exploratory data analysis and visualization of user-uploaded loop caller results, extending the capabilities beyond what Robin natively provides.

For example, in our case study, we instructed the AI assistant to perform the following analyses: create a line graph comparing loop size to resolution and average loop size, generate a line graph of the loop sizes, and produce a plot of the top two loop sizes, among others. Once the code for these instructions was generated, we utilized Robin’s built-in editor to review and refine the code as needed. After completing this review, we submitted the code within Robin to produce the requested figures. The backend mechanics of the LLM system are detailed in the LLM system section.

We have provided comprehensive documentation and an example job on the Robin website to assist users in using the web server.

## Discussion

Robin represents a significant advancement in chromatin loop caller analysis, offering a readily accessible platform complete with analysis metrics and a user-friendly web server. Users can leverage Robin-generated analysis plots, thereby preserving plot quality and accessing standard and scalable benchmarking metrics as extended from Chowdhury et al. (2024) [11]. The integration of HiGlass visualization tools within Robin facilitates the comparison of ChIP signals for assessing consistency among tools.

To ensure long-term sustainability and reproducibility, we designed Robin as a comparative analysis platform rather than a loop-calling execution environment. This decision keeps the system lightweight, focused, and easier to maintain. Instead of embedding loop-calling tools directly into the server, we provide Docker containers and associated scripts that allow users to run these tools locally and upload the resulting outputs to Robin for downstream comparative visualization and analysis. Detailed instructions for setting up and using the Docker environment are included in the Data and Code Availability section. Currently, Robin supports uploaded files up to ~100 megabytes (MB), which is significantly larger than typical loop-caller output files, usually ranging from a few hundred kilobytes to a few MB. This generous limit ensures users can upload multiple results or larger outputs without encountering file size issues, enhancing usability and flexibility.

Additionally, the LLM integration expands the scope of Robin’s utility for users. Through the AI assistant, users can effortlessly generate plots not provided by Robin with a single sentence, utilizing the same dataset. Furthermore, Robin provides users with interactive plots and the option to download high-resolution plots. Alternatively, users can also download the analysis files and use them to generate their preferred visualization plots on any GenAI platform of their choice, thus providing more flexibility.

In addition to generating Python code for data visualization, the AI assistant in Robin is capable of performing other tasks, such as providing explanations for observed patterns in analysis results. These explanations are embedded as comments within the generated code, helping users better understand the rationale behind certain visualizations or analytical choices. However, while such interpretive support is possible, we intentionally avoid making it a central feature. Interpretation via LLMs can introduce the risk of overgeneralization or misleading correlations, especially without context-specific domain supervision. We, therefore, advise users to treat these insights as supportive rather than definitive and to verify them within the appropriate biological or computational context.

Overall, Robin offers an intuitive and robust analysis platform for loop caller algorithms, streamlining the research process.

## Supplementary Material

lqag009_Supplemental_File

## Data Availability

We used Human Lymphoblastoid (GSE63525) cells to generate the example use cases included on Robin’s example page. All source code and data used in this study are available at https://doi.org/10.5281/zenodo.18227121.
